# Narrative or self-feeling? A historical note on the biological foundation of the “depressive situation”

**DOI:** 10.3389/fpsyg.2014.00009

**Published:** 2014-01-27

**Authors:** Lara Rzesnitzek

**Affiliations:** Institute History of Medicine, Universitätsmedizin CharitéBerlin, Germany

**Keywords:** depression, history of psychiatry, depersonalization, self-feeling, self-awareness

In her paper “The depressive situation” Jacobs (2013) stresses the role of normative evaluative and narrative processes in what creates the lived experience of depression. These “evaluative dynamics” are seen to emerge out of an “interplay of pre-reflective and reflective processes,” which are claimed as being “significantly altered in depression.” Even if acknowledging a pre-reflective level of the processes of evaluative self- and world-disclosure, Jacobs argues that this “cannot be reduced to or equated with a mere affective (or: “felt”) dimension.” Jacobs' philosophical analysis ties in a way on the cognitive interpretation of depression that has prevailed since the 60s especially due to the work of Aron Beck and the development of cognitive psychotherapy for depression (Beck, 1975, 1979; Clark et al., 1999). However, Jacobs well admits that affectivity provides a mode of an evaluative process.

Interestingly - and in contrast to Jacob's theory -, this *affective*, felt, dimension had once been considered as absolutely crucial for the “depressive situation.” But this was long before the cognitive turn in psychology overwhelmed psychiatry.

So, my comment just wants to remind of the role of affectivity, or more precisely what was called “self-feeling,” in the very early theories of depression and mental disorder that were formulated at the beginnings of psychiatry as a medical discipline.

## Self-feeling as gemeingefühl

The English term “*self-feeling*” was introduced into psychiatry by Alexander Crichton in his “Inquiry into the Nature and Origin of Mental Derangement” as a translation of the German “physiological” notion “‘Selbst-gefühl’ and ‘Gemeingefühl”’ (Crichton, 1798, p. 113). “*Physiological*,” because in Johann Christian Reil's theory of “Gemeingefühl” as the somatic foundation of self-consciousness, mood, temper, volition and behavior - “corresponding exactly to the feelings that we call the temperament of persons” - self-feeling was thought of as emerging primarily only from the physiological arrangement of the nervous system (Reil, 1794/1817, pp. 83–84).

## Self-feeling as selbstgefühl

The philosophical notion “Selbstgefühl” had already been introduced by Johann Bernhard Basedow in 1764 with reference to Locke as the translation of “inner sense” (Drüe, 1994) and was embraced with enthusiasm in philosophical discussions in the 1770 about self-consciousness and its relation to some sort of inner sense – crystallizing within a single notion the romantic idea of the *primary importance of feeling* (Frank, 2002). The *affective structure of self-feeling* in contrast to pure somatic perceptual or, on the contrary, the cognitive meaning of self-feeling as inner sense was repeatedly stressed.

Self-feeling was thought as the most basic and primordial sense of subjectivity, as the direct experience of existence: a feeling of being, an existential feeling. In this line, the term “Selbstgefühl” had also been chosen as the translation of the French “*sentiment de soi-même*,” a feeling inseparable of the feeling of existence, “*sens intime de l'existence*” or sentiment de notre propre existence as it was called also by Rousseau and the Encyclopaedists (Frank, 2002, p. 79f). Remarkably, both these notions (“feelings of being” and “existential feeling”) are today used by phenomenologist Matthew Ratcliffe especially in reference to a phenomenological analysis of depression (see Ratcliffe, 2008; Slaby and Stephan, 2008).

Moreover, romantic philosophers stressed the relation of “Selbstgefühl” to the latin sensus sui ipsius that, in the stoic tradition, denotes a self-feeling that every sentient living being has by nature as an integral affective component of its striving for self-preservation. In its very striving, an animal displays a background orientation towards its own life as something worth preserving (Frank, 2002, p. 28).

## Self-feeling, physiology, and mental disorder

Even if there was a vivid debate between philosophers, romantic physicians and psychiatrists concerning the correct theoretical explication and especially the *biological* basis of self-feeling, it was seen mostly as layered in itself, Gemeingefühl being the lowest most primitive form (Burdach, 1828, p. 166). In its higher levels, self-feeling was to include such background longlasting feelings as mood and temper. In this line, the 1845 textbook “Principles of Medical Psychology” - written by the romantic psychiatrist Ernst Freiherr von Feuchtersleben who coined in this context the term “psycho-somatic” - explains:

“Self-feeling unifies sensation and representation. In self-feeling, subjectivity permeates the organic body; it is what we mean when saying “I” and it provides the ground for all other feelings and emotions. Within self-feeling, the Gemeingefühl takes on a human character. The pleasantness of the latter is transformed into cheerfulness, displeasure into sadness, the changing and interplay of these states is called mood and so it comes that human weal and woe exist trough this channel of inner life.” (von Feuchtersleben, 1845, pp. 137–138).

## Self-feeling and depression

Introduced by romantic psychiatry, the idea of self-feelings being diminished in depression had been central to early German and French psychiatry.

Already in the first edition of his psychiatric textbook, “The Pathology and Therapy of Mental Diseases,” Wilhelm Griesinger noted that “especially melancholic's” complain of a “kind of anaesthesia: I see, I hear, I feel, they say, but the object does not reach me; I cannot receive the sensation; it seems to me as if there was a wall between me and the external world”(Griesinger, 1845, p. 67). Griesinger thought this form of emotional anaesthesia to be due to an “anomaly of Gemeingefühl” (Griesinger, 1845, p. 65).

In early German and French psychiatry, changes in self-feeling were seen as the core of melancholia or lypémanie, the conceptual precursors of depression. Reporting about patients that “complained almost in the same terms of a lack of sensations, […] to them it was a total lack of feelings, as if they were dead,” Albert Zeller had explained their symptoms as a disorder “self-awareness,” of “Gemeingefühl” or “self-feeling” (Zeller, 1838, pp. 522–525).

Friedrich Schäfer classified similar observations as a subtype of melancholia, naming this subtype *Melancholia anaesthetica*: “when these patients complain about their suffering, they relate it explicitly to a sort of emptiness, hollowness in their head, or in the pit of their stomach; of a discomfort of not reaching the surroundings with their inner selves. They see and hear everything, but without experiencing any representation or feeling of their inner stirrings, of their sensory vividness” (Schäfer, 1880, p. 242).

Published in the same year as Feuchtersleben's textbook, also Gotthilf Heinrich Schubert's “Diseases and Disorders of the Human Soul” explained, that it would be “the self-feeling of the melancholic that is tarnished and impaired” – a distortion that robs the melancholic of every energy to entertain any wantings, and this while having full insight into his own misery;” a state that Schubert called “paralyzing melancholia” (Schubert, 1845, p. 303).

In France, Jean-Étienne Esquirol blew long in the very same horn: “An abyss, they say, separates them from the external world, I hear, I see, I touch, say many lypemaniacs, but I am not as I formerly was. Objects do not come to me, they do not identify themselves with my being; a thick cloud, a veil changes the hue and aspect of objects” (Esquirol, 1838, p. 414).

Taking a description of a similar state in Amiel's “Journal Intime,” Ludovic Dugas coined the term “*depersonalization*” in 1898, initiating a rich debate about the correct phenomenological description, classification and biological underpinnings of these symptoms of emotional numbing, anomalous body experience, anomalous subjective recall and alienation from one's surroundings (Dugas and Moutier, 1911; Sierra and Berrios, 1997).

In the light of the complaints so often uttered by depressed patients that they “feel nothing,” that they are no more than “a corpse,” or even to “be dead,” it was Jules Cotard who especially focused on these symptoms of anomalous corporal experience - ranging from somatosensory distortions, feelings of disembodiment up to the loss of body ownership feelings - in his 1880 lecture “On hypochondriacal delusions in a severe form of anxious melancholia,” later on called “délire de negation” – délire however being a complex of emotional, volitional and intellectual symptoms and not a delusion in the sense of a false idea or thought or disordered thinking (Cotard, 1880, 1882).

Findings of modern neuropsychiatric research also speak for a *biological foundation of these symptoms*, probably involving a down regulation of the amygdale because of stress induced hyperactivity in the orbito-frontal cortex (Sierra and Berrios, 1998). Whatever the complete and correct biological explanation of the impairment of self-feeling seen in the emotional numbing and the related inability to color experience with feelings, these affective changes in depressive illness seem to pertain to a very basic affective dimension of self-feeling, as many autobiographic patient narratives and memoirs illustrate in drastic terms (see, e.g., Styron, 1990; Kuiper, 1995; Thompson, 1996; Solomon, 2001; Brampton, 2008; see also Ratcliffe, 2008).

The utter strangeness and distance from everyday experience of these symptoms encountered in severe melancholic depression is what can present a real challenge in treating depression. Such a complete loss of emotional colouring – a colouring taken for granted in our everyday lives – is almost impossible to imagine. Moreover, it seems not to be a result of a narrative or cognitive evaluation. Consequently, it cannot, so to say, be discussed away. Therefore, these severe depressive symptoms might remain quite resistant to psychotherapy, especially cognitive psychotherapy, but show most often relievable by somatic therapies as medication or electro-convulsive therapy (APA, 2001, 2010).

## Conclusion

Romantic psychiatry knew of a notion of “self-feeling” as referring to a pre-reflective *affective* form of self-awareness that is the foundation of a person's mood and temper and with these of her motivational, evaluative and practical perspective on the world (vgl. Slaby, 2012). In contrast to its remote ramifications with terms such as “self-confidence” or “self-esteem,” “self-feeling” also encompassed the opposite of self-confidence, i.e., states of low self-esteem, self-depreciation, submissiveness and the like and was thought to originate in a very basic somatic organismic dimension: a quite basic feeling of vitality or “vital tone” of a person's existence. A feeling so basic and seen as granted that only loss of or change in it - as in depression - might remind of its existence.

## References

[B1] American Psychiatric Association, Practice Guidelines. (2010). Treatment of Patients with Major Depressive Disorder. Available online at: http://psychiatryonline.org/guidelines.aspx.

[B2] American Psychiatric Association, Task Force on Electroconvulsive Therapy. (2001). The Practice of Electroconvulsive Therapy: Recommendations for Treatment, Training, and Privileging. Washington, DC: American Psychiatric Association

[B3] Brampton S. (2008). Shoot the Damn Dog: A Memoir of Depression. London: Bloomsbury

[B4] Beck A. T. (1975). Cognitive Therapy and the Emotional Disorders. New York, NY: Intl Universities Press

[B4a] Beck A. T. (1979). The Cognitive Therapy of Depression. New York, NY: Guilford Press

[B5] Burdach K. F. (1828). Die Physiologie als Erfahrungswissenschaft. Band *2*, Leipzig: Leopold Voß

[B6] Clark D. A. Beck A. T. Alford B. A. (1999). Scientific Foundations of Cognitive Theory and Therapy of Depression. New York, NY: Wiley

[B7] Cotard J. (1880). Du Délire hypochondriaque dans une forme grave de la mélancholie anxieuse. Ann. Méd. Psychol. 38, 168–174

[B8] Cotard J. (1882). Du Délire des négations. Archives de Neurologie 4, 152–170 and 282–295. 7625193

[B9] Crichton A. (1798) An Inquiry into the Nature and Origin of Mental Derangement. London: T. Cadell jr. and W. Davies 10.1177/1087054708315137 18936239

[B10] Drüe H. (1994). Die Entwicklung des Begriffs Selbstgefühl in Philosophie und Psychologie. Archiv für Begriffsgeschichte 37, 285–305

[B11] Dugas L. Moutier F. (1911). La Dépersonnalisation. Paris: Felix Alcan

[B12] Esquirol J. E. (1838). Des Maladies Mentales Considerées Sous Les Rapports Médical, Hygiénique et Médico-Légal. Tome 1, Paris: J. B. Baillière PMC5093814 29918508

[B13] von Feuchtersleben E. F. (1845). Lehrbuch der ärztlichen Seelenkunde. Wien: C. Gerold

[B14] Frank M. (2002). Selbstgefühl. Frankfurt/M: Suhrkamp

[B15] Griesinger W. (1845). Die Pathologie und Therapie der psychischen Krankheiten. Suttgart: Krabbe

[B16] Jacobs K. A. (2013). The depressive situation. Front. Psychol. 4:429 10.3389/fpsyg.2013.00429 23882238 PMC3713339

[B17] Kuiper P. C. (1995). Seelenfinsternis. Die Depression eines Psychiaters. Frankfurt: Fischer

[B18] Ratcliffe M. J. (2008). Feelings of Being. Phenomenology, Psychiatry, and the Sense of Reality. Oxford: Oxford University Press 10.1093/med/9780199206469.001.0001

[B19] Reil J. C. (1794/1817). Vom Gemeingefühl. Halle 1794, Reprinted in: J. C. Reil's kleine Schriften. Halle: Verlag der Curtschen Buchhandlung

[B20] Schäfer F. (1880). Bemerkungen zur psychiatrischen Formenlehre. Allg. Z. Psychiat. 36, 214–278

[B21] Schubert G. H. (1845). Die Krankheiten und Störungen der menschlichen Seele. Stuttgart und Tübingen: J. G. Cotta

[B22a] Sierra M. Berrios G. E. (1997). Depersonalisation: a conceptual history. Hist. Psychiatry 8, 213–229 11619439 10.1177/0957154X9700803002

[B22] Sierra M. Berrios G. E. (1998). Depersonalization: biological perspective. Biol. Psychiat. 44, 898–908 10.1016/S0006-3223(98)00015-8 9807645

[B23] Slaby J. (2012). Affective self-construal and the sense of ability. Emot. Rev. 4, 151–156 10.1177/1754073911430136

[B24] Slaby J. Stephan A. (2008). Affective intentionality and self- consciousness. Conscious. Cogn. 17, 506–513 10.1016/j.concog.2008.03.007 18417362

[B25] Solomon A. (2001). The Noonday Demon: An Atlas of Depression. London: Vintage

[B26] Styron W. (1990). Darkness Visible. London: Vintage

[B27] Thompson T. (1996). The Beast: A Journey through Depression. New York, NY: Plume

[B28] Zeller A. (1838). Ueber einige Hauptpunkte in der Erforschung und Heilung der Seelenstörungen. Zeitschrift für die Beurtheilung und Heilung der krankhaften Seelenzustände 1, 515–569

